# Analysis on the MRI and BAEP Results of Neonatal Brain With Different Levels of Bilirubin

**DOI:** 10.3389/fped.2021.719370

**Published:** 2022-01-31

**Authors:** Zhongxing Lu, Shouling Ding, Fen Wang, Haitao Lv

**Affiliations:** ^1^Children's Hospital, Soochow University, Suzhou, China; ^2^Neonatology Department, Changzhou Maternal and Child Health Care Hospital, Changzhou, China; ^3^Pediatrics Department, Taicang First People's Hospital, Changzhou, China

**Keywords:** MRI abnormality change, acute bilirubin encephalopath, bilirubin, hyperbilirubinemia, BAEP

## Abstract

**Background:**

To explore whether there is abnormality of neonatal brains' MRI and BAEP with different bilirubin levels, and to provide an objective basis for early diagnosis on the bilirubin induced subclinical damage on brains.

**Methods:**

To retrospectively analyze the clinical data of 103 neonatal patients, to conduct routine brain MRI examination and BAEP testing, and to analyze BAEP and MRI image results of the neonatal patients, who were divided into three groups based on the levels of total serum bilirubin concentration (TSB): 16 cases in mild group (TSB: 0.0–229.0 ěmol/L), 49 cases in moderate group (TSB: 229.0–342.0 ěmol/L), and 38 cases in severe group (TSB ≥ 342.0 ěmol/L).

**Results:**

We found the following: A. Comparison of the bilirubin value of the different group: The bilirubin value of the mild group is 171.99 ± 33.50 ěmol/L, the moderate group is 293.98 ± 32.09 ěmol/L, and the severe group is 375.59 ± 34.25 ěmol/L. The comparison of bilirubin values of the three groups of neonates (*p* < 0.01) indicates the difference is statistically significant (*p* < 0.01). B. The weight value of the <2,500 g group is 2.04 ± 0.21 and the ≥2,500 g group is 3.39 ± 0.46; the weight comparison of the two groups indicates that the difference is statistically significant (*p* < 0.01). C. Comparison of the abnormal MRI of the different groups: The brain MRI result's abnormal ratio of the mild group is 31.25%, the moderate group is 16.33%, and the severe group is 21.05%, but the comparison of brain MRI results of the three neonates groups indicates that the difference is not statistically significant (*p* > 0.05). D. Comparison of abnormal MRI signal values of globus pallidus on T1WI in different groups: 1. The comparison of normal group signal values with that of mild group (*p* < 0.05), with that of moderate group, and with that of severe group (*p* < 0.01) indicates that the difference is statistically significant.

**Conclusion:**

At low level of bilirubin, central nervous system damage may also occur and can be detected as abnormality by MRI and BAEP. Meanwhile, MRI and BAEP can also provide early abnormal information for the judgment of central nervous system damage of the children with NHB who have no acute bilirubin encephalopathy (ABE) clinical features, and provide clues for early treatment and early intervention.

## Highlights

- In patients without ABE, bilirubin neurological damage may occur at low levels of bilirubin, and the abnormal rate of MRI does not increase with the increase of bilirubin levels. Craniocerebral MRI abnormal changes and no abnormal changes in jaundice patients showed no statistical difference in bilirubin levels.
- In patients with jaundice without bilirubin encephalopathy, bilirubin-induced abnormal brain MRI changes and body mass, gestational age, and bilirubin levels showed no significant correlation.

## Background

Neonatal jaundice is quite common, affecting 60–80% of newborns overall (1). With high accumulation of unconjugated bilirubin, severe patients (their bilirubin's concentration >20 mg/dl) may suffer from bilirubinencephalopathy or kernicterus, and bilirubun-induced neurological dysfunction (BIND) (2), which will cause permanent damage to the basal ganglia, hippocampus, hypothalamus, cerebellar neurons, and brainstem (3, 4). Clinical manifestation includes lethargy, poor response, fever, apnea, convulsion, auditory complications, and so on (2). Magnetic resonance imaging (MRI) and hearing screening have proved to be highly sensitive in bilirubin encephalopathy. With MRI, high intensity signals of globus pallidus and subthalamic nucleus can be detected in early T1WI, and high stripy symmetric signals of globus pallidus can be detected in later T2WI (5, 6). Abnormal changes occur in the BAER (7). However, severe hyperbilirubinemia (>20 mg/dl) that could potentially lead to bilirubin encephalopathy or kernicterus and neurodevelopmental complications is said to be much rarer, affecting <2% of newborn infants (8); most of them have no clinical manifestation except jaundice. It is still debated whether these lower levels of hyperbilirubinemia cause brain damage to neonates or not. It is also unclear whether different degrees of hyperbilirubinemia result in different patterns of brain injury. To answer these questions, we examined the brain MRI and hearing screening of neonates with different levels of bilirubin levels.

## Methods

### Methods and Criteria

#### Methods

MRI: A Siemens Skyra 3.0T superconductivity magnetic resonance scanner and new Tim4G platform equipped with 20 units of high-density matrix head and neck joint coils were used for this study. Before MRI scanning, the neonate patients were given routine sedation and kept in supine position and warm. T1WI (TR = 477 ms, TE = 15 ms), T2WI (TR = 4,060 ms, TE = 120 ms), matrix 256 × 256, slice thickness 5 mm, gap 0.4 mm. Images were obtained, and coronal scanning was done for all neonate patients.

#### Criteria for Diagnosis

All MRI results were jointly written and analyzed by two radiologists who did not know the clinical history of patient.

MRI: High intensity signals of globus pallidus and subthalamic nucleus detected in T1WI, or high intensity symmetric signals of globus pallidus detected in T2WI or uneven signal of basal ganglion were considered abnormal. No abovementioned manifestation was regarded as normality (5, 6).

#### Brainstem Auditory Evoked Potential

BAEP testing was performed by an otolaryngologist skilled in BAEP technology in a quiet environment.

#### Criteria for Diagnosis

BAEP: The results were considered to be abnormal as shown that the incubation period of I wave, III wave, and V wave is prolonged, the interphase period of I ~ V wave is prolonged, the waveform is poorly differentiated or disappears, and the single or bilateral I wave, III wave, and V wave all disappear (7).

### Statistical Method

Data analysis was performed by the use of SPSS 23.0 statistical software. Each measuring parameter was expressed by mean ± SD and *t*-tests (multi-group comparisons), while each counting parameter was expressed by rate and *c*^2^ tests (comparisons among groups). *P* < 0.05; the difference is statistically significant.

## Results

### Clinical Data

We retrospectively analyzed 103 neonates (51 boys and 52 girls), in which we assume that these neonates have no “clinical” manifestation of bilirubin encephalopathy and had been hospitalized in Neonatology Department of Taicang First People's Hospital from March 2013 to September 2015. Their gestational ages ranged from 31 + 6 weeks to 43 + 1 weeks (in average,38.14 ± 2.21 weeks), birth weight from 1.55 to 5.00 kg (average, 3.18 ± 0.64 kg), 26 pre-term neonates, and 77 full-term neonates. These neonates were divided into three groups based on their levels of serum bilirubin concentration: 16 cases in mild group (TSB: 0.0–229.0 ěmol/L), 49 cases in moderate group (TSB: 229.0–342.0 ěmol/L), and 38 cases in severe group (TSB ≥ 342.0 μmol/L). Possible diseases which have manifestations similar to that of bilirubin encephalopathy, such as toxic cerebral hypoxia damage, hepatolenticular degeneration, hypoxic ischemic encephalopathy, intracranial hemorrhage, and hypoglycemia, were excluded.

#### Comparison of the Bilirubin Value of the Different Group

1. The bilirubin value of the mild group is 171.99 ± 33.50 ěmol/L, the moderate group is 293.98 ± 32.09 ěmol/L, and the severe group is 375.59 ± 34.25 ěmol/L. The comparison of bilirubin values of the three groups of neonates (*p* < 0.01) indicates that the difference is statistically significant. 2. The bilirubin value of the pre-term group is 289.70 ± 85.38 ěmol/L and the full-term group is 310.36 ± 72.32 ěmol/L, but the comparison of the bilirubin values between pre-term group and full-term group (*p* > 0.05) indicates that the difference is not statistically significant. 3. The bilirubin value of the normal brain MRI group (82) is 305.55 ± 74.54 ěmol/L, and the abnormal brain MRI group is 303.56 ± 83.04 ěmol/L; The comparison of bilirubin values between the two groups (*p* > 0.05) indicates that the difference is not statistically significant (Table 1).

**Table 1:** Comparison of the bilirubin value of the different group.

**Group**	**Bilirubin value**	***t***	***p***
Mild group	171.99 ± 33.50	20.54	0.00
Moderate group	293.98 ± 32.09	64.13	0.00
Severe group	375.59 ± 34.25	67.59	0.00
Pre-term	289.70 ± 85.38	−1.20	0.232
Full-term	310.36 ± 72.32		
Normal MRI	305.55 ± 74.54	0.11	0.92
Abnormal MRI	303.56 ± 83.04		

#### Two Groups Divided Based on the Weight of 2,500 g

The brain MRI result's abnormal ratio of the mild group is 31.25%, the moderate group is 16.33%, and the severe group is 21.05%, but the comparison of brain MRI results of the three neonates groups (*p* > 0.05) indicates that the difference is not statistically significant (Table 2).

**Table 2:** Two groups divided based on the weight of 2,500 g.

**Group**	**<2,500 g**		**≥2,500 g**
Weight	2.04 ± 0.21		3.39 ± 0.46
*T*		−18.85	
*P*		0.00	

*The weight value of the <2,500 g group is 2.04 ± 0.21 and the ≥2,500 g group is 3.39 ± 0.46; the weight comparison of the two groups indicates that the difference is statistically significant (p <0.01)*.

#### Comparison of the Abnormal MRI of the Different Groups

The brain MRI result's abnormal ratio of the pre-term is 30.77% and the full-term group is 16.88%, but the comparison of brain MRI results between prem-term group and full-term group (*p* > 0.05) indicates that the difference is not statistically significant. The brain MRI result's abnormal ratio of the <2,500 g group is 37.50% and the ≥2,500 g group is 17.24%, but the comparison of brain MRI results of two neonates groups (*p* > 0.05) indicates that the difference is not statistically significant (Table 3).

**Table 3:** Comparison of the abnormal MRI of the different groups.

**Group**	**Normal MRI**	**Abnormal MRI**	**c^**2**^**	***p***
Mild group	11 (68.75%)	5 (31.25%)		
Moderate group	41 (83.67%)	8 (16.33%)	1.579	0.454
Severe group	30 (78.95%)	8 (21.05%)		
Pre-term	18 (69.23%)	8 (30.77%)	2.310	0.160
Full-term	64 (83.12%)	13 (16.88%)		
<2,500 g	10 (62.50%)	6 (37.50%)	3.420	0.09
≥2,500 g	72 (82.76%)	15 (17.24%)		

#### Comparison of Abnormal MRI Signal Values of Globus Pallidus on T1WI in Mild, Moderate, and Severe Groups and Comparison of These Abnormal MRI Signal Values With Normal MRI Signal Values

1. The comparison of normal group signal values with that of mild group (*p* < 0.05), with that of moderate group, and with that of severe group (*p* < 0.01) indicates that the difference is statistically significant. 2. The comparison of signal values between mild and moderate groups (*p* < 0.05) and between mild group and severe group (*p* < 0.01) indicates that the difference is statistically significant. 3. The comparison of signal values between moderate group and severe group (*p* < 0.05) indicates that the difference is statistically significant (Table 4).

**Table 4:** Comparison of abnormal MRI signal values of globus pallidus on T1WI in mild, moderate, and severe groups.

**Group**	***n***	**Mean signal value of left globus pallidus**	**Mean signal value of right globus pallidus**
Normal group	82	892.03 ± 132.54	878.65 ± 126.43
Mild group	5	956.85 ± 245.87	942.06 ± 232.60
Moderate group	8	1,056.23 ± 254.21	1,032.32 ± 222.32
Severe group	8	1,246.03 ± 278.46	1,239.03 ± 288.53

#### Comparison of BAEP Testing Results for Neonate Patients With Abnormal MRI in Three Groups and Comparison of Hearing Screening Results Between Neonate Patients With Abnormal MRI and Normal MRI

1. There were 27 cases in abnormalities of the BAEP results of all 103 bilirubin patients. 2. There were 15 cases in abnormalities of the BAEP result of the 82 cases of normal brain MRL, two cases in abnormalities of the BAEP result of the five cases of abnormal MRI in mild bilirubin group, four cases in abnormalities of the BAEP result of the eight cases of abnormal MRI in moderate bilirubin group, and six cases in abnormalities of the BAEP result of the eight cases of abnormal brain MRI in severe bilirubin group. 3. After 1 month review of the BAEP result, there was 0 abnormal case in the normal MRI and the mild group; there were one abnormal case in the moderate group and two cases in the severe group (Table 5).

**Table 5:** Comparison of BAEP testing results for neonate patients with abnormal MRI in three groups and comparison of hearing screening results between neonate patients with abnormal MRI and normal MRI.

**Group**	**Normal MRI (82)**	**Abnormal MRI in mild group (5)**	**Abnormal MRI in moderate group (8)**	**Abnormal MRI in severe group (8)**
Normal	67	3	4	2
Abnormal	15	2	4	6
%	18.29	40.00	50.00	75.00
Review abnormality after 1 month	0	0	1 (12.50)	2 (25.00)

### MRI Pictures

A. A newborn with birth weight of 3.05 kg, with normal delivery at 36 + 3 weeks and clear amniotic fluid, Apgar score of 1 '-9', 5 '-9'. The serum total bilirubin was 502.3 mol/L on the 6th day after birth. Routine MRI of the basal ganglia examined on the 9th day after birth showed equal signal or slightly higher signal on T1WI, and the signal of globus pallidus was slightly higher than other nuclei in the basal ganglia on T1WI.

B. A newborn with birth weight of 3.50 kg, with normal delivery at 40 + 4 weeks and amniotic fluid III pollution, Apgar score of 1'-9', 5 '-9'. The serum total bilirubin was 130.4 mol/L on the 8th day after birth. Routine MRI of the basal ganglia examined on the 9th day after birth showed high signal on T1WI, and the signal of globus pallidus was symmetrical high signal on T1WI.

C. A newborn with birth weight of 3.25 kg, with normal delivery at 37 + 6 weeks and clear amniotic fluid, Apgar score of 1 '-8', 5 '-9'. The serum total bilirubin was 278.3 mol/L on the 5th day after birth. Routine MRI of the basal ganglia examined on the 8th day after birth showed high signal on T1WI, and the signal of globus pallidus was symmetrical high signal on T1WI.

D. A newborn with birth weight of 2.87 kg, with normal delivery at 38 + 5 weeks and clear amniotic fluid, Apgar score of 1 '-8', 5 '-8'. The serum total bilirubin was 478.6 mol/L on the 7th day after birth. Routine MRI of the basal ganglia on the 10th day after birth showed high signal on T1WI, and the signal of globus pallidus was symmetrical high signal on T1WI.

**Figure 1:**
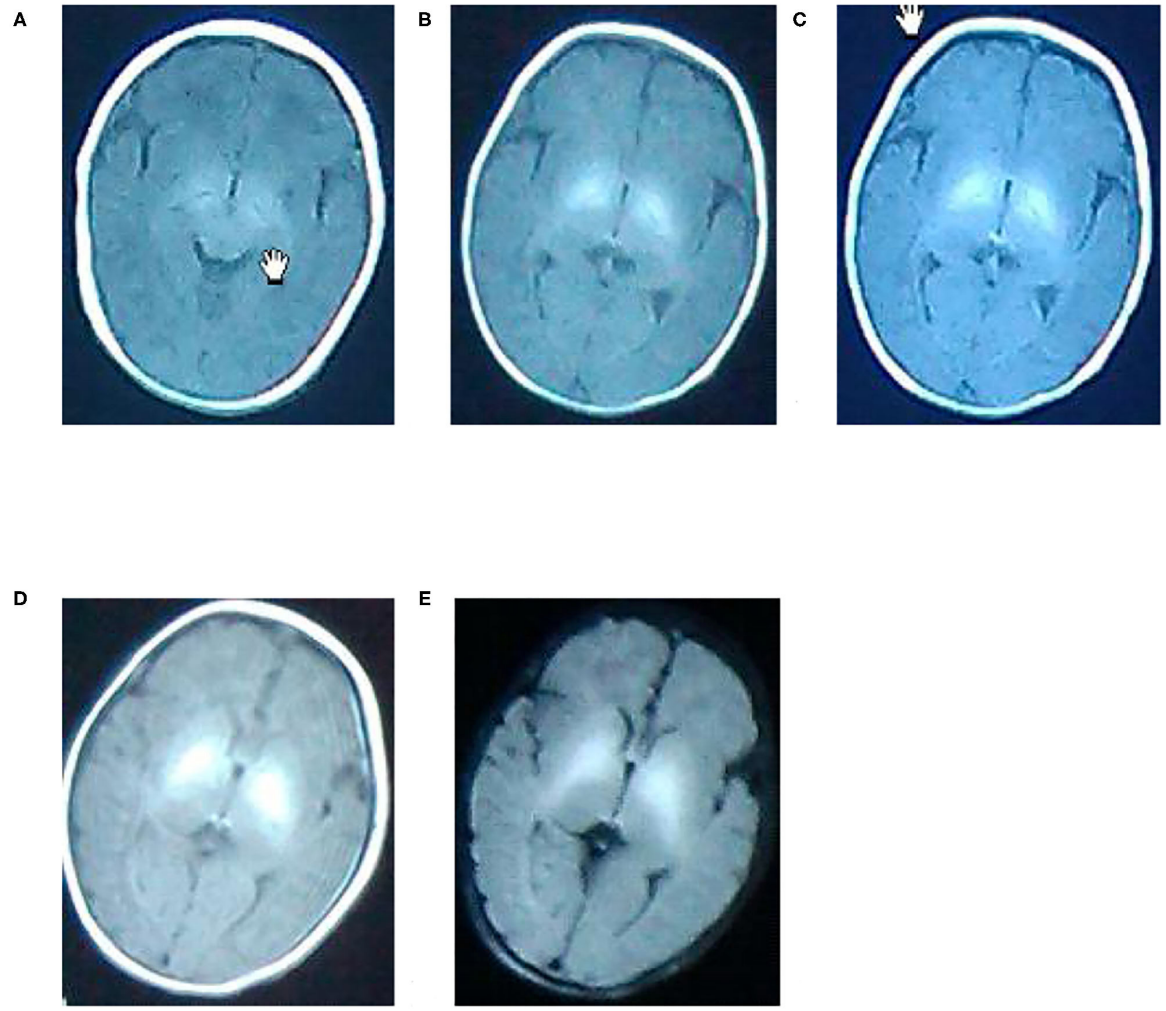
Normal **(A)**, mild **(B)**, moderate **(C)**, and severe **(D,E)** T2WI manifestation.

E. A newborn with birth weight of 3.31 kg, with normal delivery at 40 weeks and clear amniotic fluid, Apgar score of 1 '-8', 5 '-9'. The serum total bilirubin was 327.3 mol/L on the 7th day after birth. Routine MRI of the basal ganglia at 10 postnatal days showed uneven signals in bilateral basal ganglia. Routine MRI of the basal ganglia revisited at 2 months showed high signals on T2WI, and the signal of globus pallidus was symmetrical high signals on T2WI, as shown in Figure 1.

## Discussion

Neonatal jaundice is the most common clinical manifestation that occurred in neonate period, which is mainly caused by the increase of serum bilirubin in neonates. In the prenatal period, due to the hypoxic environment, the amount of red cells is increased and the hemoglobin content in the fetal is high. After birth, due to the hyperoxic environment, the survival time of red blood cell becomes short. The fetal hemologlobin is gradually converted to adult hemoglobin producing a large amount of bilirubin, which exceeds the ability of liver to treat bilirubin. When bilirubin levels are above 139 ěmol/L, skin or organ xanthochromia may appear, that is called neonate jaundice. It has been previously thought if the concentration of bilirubin in the blood is above 342 ěmol/L, serious damage to nervous system for the neonates may occur and the acute stage manifestation of acute bilirubin encephalopathy (ABE) or chronic kernicterus and BIND appears (1, 9). Brain damage caused by neonatal hyperbilirubinemia (NHB) is not always reversible and may lead to cerebral palsy and hearing loss. The most common types of nerve injuries are the sequelae of severe neurological dyskinesia and hearing impairment (2, 10–12). Acute bilirubin encephalopathy or kernicterus and BIND mainly affects subcortical regions, such as globus pallidus, hypothalamus, substantia nigra, cerebellar dentate nucleus, hippocampus, or brainstem (13, 14). The underlying mechanisms are still unknown (15). Most ABE neonatal patients still have a chance to recover by timely treatment of reducing serum bilirubin levels, while only a few suffer from sequelae, like kernicterus.

The feature of MRI manifestation to bilirubin encephalopathy is the high signal of globus pallidus in the acute stage of T1-weighted imaging (T1WI). With the development of the disease, it has changed from the high signal in acute stage of T1WI to the high symmetric signal of bilateral globus pallidus and subthalamic nucleus in chronic stage and FLAIR sequence signal (16–18). Since in the neonatal period the basal ganglia nerve cells have strong physiological and biochemical metabolism and the oxygen consumption is increasing, especially, the middle and later part of the globus pallidus is the most sensitive (18). This leads to the selective deposition of serum bilirubin in the globus pallidus, and the middle and later part of the globus pallidus is more sensitive (18–20). Besides, it will cause the damage to neurons and glial cells, the apoptosis of neurons, and the change of glial cells' mitochondria function (21, 22). Basal ganglia pallidus injury caused by hyperbilirubinemia can be efficiently detected by MRI. The high and symmetric signal of bilateral T1WI pallidal is an important imaging feature of neonatal ABE, while the change from high signal of T1WI to high signal of T2-weighted imaging (T2W1) is the imaging feature of nuclear jaundice on MRI, indicating neuronal cell necrosis and poor prognosis (23). Presently, the mechanism of MRI signal change still remains unclear. This may be related to the reduction of the T1 value, which is caused by the deposition of bilirubin in glial cells and the destruction of bilirubin to nerve cell membrane (24).

Most of the MRI results mentioned above were obtained from brain MRI findings of the severe or profound NHB patients with bilirubin encephalopathy or non-bilirubin encephalopathy. So far, there is no report about whether the MRI results of mild and moderate NHB patients without bilirubin encephalopathy manifestation are abnormal. Therefore, this study retrospectively analyzes the bilirubin level and MRI results of 103 patients with jaundice in order to understand whether there is abnormal brain MRI at different bilirubin levels, as well as whether there is statistical difference of abnormal brain MRI results in bilirubin levels, gestational age, and birth weight.

It is reported in literature that some mild levels of bilirubin could cause temporary or permanent neurological sequelae under the condition that a certain level of bilirubin is considered as being safe by people (25). Based on total serum bilirubin concentration (TSB), 103 patients in our research were divided into three groups, including 16 cases in the mild group (TSB:0.0–229.0 ěmol/L), 49 cases in the moderate group (TSB: 229.0–342.0 ěmol/L), and 38 cases in the severe group (TSB ≥ 342.0 ěmol/L). There were 21 cases with abnormal MRI results, which consisted of 5 (31.25%) cases in mild group, 8 (16.33%) cases in moderate group, and 8 (21.05%) cases in severe group (Table 3). The comparison of TSB among the three groups indicated that the difference was statistically significant (*p* < 0.01) (Table 1), whereas the comparison of abnormal brain MRI results among the three groups indicated that the difference was not statistically significant (*p* > 0.05). This pointed out that bilirubin brain nerve damage may occur in patients without the manifestation of ABE even in low level of bilirubin, and the rate of MRI abnormality is not rising with the rising of bilirubin level. The study findings are not consistent with the reports of EI Houchi et al. that the higher the total bilirubin level is, the higher the proportion of abnormal MRI is (26). We think that low number of cases in the mild group may also be an important factor. Under Taoka et al.'s follow-up of the observed subjects for 2 years, it was found that the infants had been developing normally whether the high symmetric T1WI signal was demonstrated in globus pallidus (GP) and substantia niga (STN) regions or not. In accordance with reports in the literature, it may be related to the development of gray matter mass in GP and STN after the birth of neonates (27–30). Besides, lots of literatures have reported that this manifestation is one of the brain MRI findings of nuclear jaundice (25, 31, 32). Along with the extensive clinical application of MRI, the manifestation is becoming more and more common in neonates and normal neonates. Thus, some scholars recently have raised different opinions (26–28). For example, Harris et al. (33) reported that high symmetric signal in the globus pallidus region of four 5–21 day neonates with acute kernicterus has disappeared during the follow-up. This manifestation was thought transient and had no correlation with the prognosis of the patients. This manifestation also appeared in the cases of neonatal hypoxic ischemic encephalopathy, hypoglycemia, and other cases (34, 35).

In this study, the neonate patients, with high signal of globus pallidus in T1WI or T2W1, caused by the diseases of neonatal hypoxic ischemic encephalopathy, hypoglicemia, hepatolenticular degeneration, and other diseases, have been excluded. Of our 21 patients with MRI, 12 were followed up for 1 month, and nine cases were followed up for 3 months. Six cases of patients had normal brain MRI results in the 1 month later reexamination. Three cases of patients had not been reexamined of the MRI, and one of the three cases had neurological abnormalities 2 months later, who were delivered with gestational age 40 weeks, birth weight 3.31 kg, clear amniotic fluid, the Apgar score 8 at 1 min and 9 in 5 min, maximum bilirubin value 327.3 ěmol/L, and uneven signal of the brain MRI in bilateral basal ganglia region. However, one case of this study showed no brain MRI abnormalities with maximum bilirubin value 502.3 ěmol/L, birth weight 3.05 kg, gestational age 36 + 3 weeks, clear amniotic fluid, and the Apgar score 9 at 1 min, 9 in 5 min. In addition, another case in the study may have ABE with gestational age 40 + 4 weeks, birth weight 3.5 kg, meconium stained amniotic fluid during birth, the Apgar score 9 at 1 min, 9 in 5 min, the maximum bilirubin value 130.4 ěmol/L, and high symmetric T1WI signal of bilateral basal ganglia indicated by his brain MRI. The high symmetric signal of bilateral globus pallidus is not unique to the neonatal ABE, and it can also be seen in some neonatal patients with hypoxic ischemic encephalopathy (19, 36) or even in normal neonates. But the MRI manifestations of hypoxic ischemic encephalopathy (HIE) involved more extensive scope, which was characterized by internal capsule, the putamen, and the thalami, and accompanied by cortical and subcortical, deep leukoplakia plaque abnormal signals, diffuse brain edema, intracranial hemorrhage, and so on. These accompanied manifestations were rarely reported in bilirubin encephalopathy (37).

Blood brain barrier injury might be caused by anoxia or other factors in antepartum and intrapartum and increased the permeability of blood brain barrier, resulting in the increasing of free bilirubin to enter into brain tissue through the injured blood-brain barrier and to deposit in the basal nerve nucleus, cerebral ganglia, subthalamic nucleus, parietal nucleus, ventricular nucleus, caudate nucleus, cerebellum, oblongata, cerebral cortex, spinal cord, and brainstem (38). Accordingly, the utilization of oxygen in brain tissue was inhibited, 176 leading to brain damage. It is also believed that bilirubin deposition causes the influx of neuron cells Ca2+ (39) and stimulates the increase of proteolytic enzyme activity (40), oxidative damage (41), immune stimulation, immunotoxicity (42), increased cytoglutamate and excitotoxicity (43), imflammatory damage (44), and other pathways, leading to neuronal necrosis and apoptosis (36). It has been reported in many studies that the occurrence of neonatal BE is related to the factors of gestational age (45), birth weight (46), bilirubin binding state, and bilirubin level (26). In this study, 103 patients were divided into preterm group (26 cases) and full-term group (77 cases), observing the comparison of bilirubin values (289.70 ± 85.38 vs. 310.36 ± 72.32, *p* = 0.232) of the two groups, and the MRI abnormal result (*p* = 0.16) of the two groups was *p* > 0.05; the difference was not statistically significant, which indicated that there may be no difference in the toxicity of bilirubin to central nervous system between pre-term group and full-term group without BE clinical manifestation. Moreover, it did not appear to be the premature infants with younger gestational age were more susceptible to bilirubin toxicity. The analyses of this result may be as follows: (1) Premature delivery patients, who are usually admitted to be in hospital after birth, are treated for jaundice in time during hospitalization. Full-term infants are admitted to be in hospital only when their bilirubin value reaches a higher level. On the one hand, high level of bilirubin could easily cause nerve damage through the blood brain barrier; on the other hand, the longer the high level of bilirubin remains in the body, the more neurotoxicity of bilirubin is (20). (2) The statistical analysis in this study may be biased, on account of the fewer cases of pre-term delivery group, especially lack of pre-term patients <31 weeks of gestational age due to obstetric factors. Meanwhile, we also observed whether there was statistical difference between birth weight and brain MRI abnormality of the jaundice patients. Based on birth weight, 103 patients were divided into two groups, one group birth weight <2,500 g (16 cases, including six abnormal cases) and the other group birth weight ≥2,500 g (87 cases, including 15 abnormal cases) (Table 2). The comparison of brain MRI abnormality of the two groups was *p* = 0.09, *p* > 0.05, the difference was not statistically significant.

In addition, the comparison of bilirubin value between abnormal MRI group and normal MRI group was (303.56 ± 83.04 vs. 305.55 ± 74.54, *p* = 0.92, *p* > 0.05) (Table 1); the difference was not statistically significant, which indicated that there was no obvious difference in bilirubin level between abnormal MRI group and normal MRI group of jaundice patients without ABE.

At the same time, we measured the T1WI signal values of the patients with abnormal brain MRI and the signal values of the patients with normal brain MRI in the three groups, and performed statistical comparison, which showed that the T1WI signal values of the patients with abnormal MRI were all higher than those of the patients with normal MRI (*p* < 0.05), and the T1WI signal values of the patients with MRI abnormalities in the three groups also had statistically difference (*p* < 0.05) (Table 4). With the increase of serum bilirubin level, the T1WI signal value of the patients with MRI abnormalities also increased, which was consistent with the report of Zhou et al. (47) that there was a linear correlation between the mean signal value of globus pallidus and serum total bilirubin levels in the lesion group. This indicated that with the rising of bilirubin level, the more bilirubin deposited on neuron such as globus pallidus, the more serious damage would be made to nerve tissue such as neuron.

NHB could not only lead to acute bilirubin encephalopathy and kernicterus but also bilirubin induced neurological dysfunction (BIND) (48, 49), which includes mild neurological abnormalities, cognitive disorder, auditory neuropathy spectrum disorder (ANSD) (50), and so on. Auditory complications, a disabling neurological finding in kernicterus, are typically characterized by varying degree of auditory neuropathy/dys-synchrony (AN/AD) ranging from central auditory processing difficulties with normal hearing to severe AN/AD with absent auditory brainstem responses, and possibly accompanying severe hearing loss and deafness. In fact, the brainstem cochlear nuclei are said to be one of the first structures affected by elevated total bilirubin, followed by the auditory nerve (3, 4). So we performed hearing tests for the 103 patients with brainstem auditory evoked potential (BAEP) (51) devices. From Table 5, it was found that 15 cases of 82 patients with normal MRI results were abnormal, accounting for 18.29%. In the mild group, two cases of five patients with abnormal MRI were abnormal, accounting for 40.00%. In the moderate group, four cases of eight patients with abnormal MRI were abnormal, accounting for 50.00%. In the severe group, six cases of eight patients with abnormal MRI were abnormal, accounting for 75.00%. The change of BAEP is closely related to bilirubin concentration and duration, but BAEP may be abnormal in children with moderate bilirubin level, that is, BAEP may be abnormal in children with related safe bilirubin ormalities that were improved after clinical treatment concentration even if there is no clinical manifestion (52, 53). BAEP was improved with the decrease of serum bilirubin or after the process intervention such as phototherapy and blood exchange transfusion (54–56). Akinpelu et al. (52). reported in 2013 that the abnormal BAEP rate fluctuated between 9 and 83.3% before clinical intervention, and about over half of the BAEP abnormalities were improved after clinical treatment. After admission, all 103 patients were treated with medication and/or phototherapy, and their jaundice decreased significantly. After 1–3 month follow-up, the reexamination found that the patients with hearing abnormalities in the MRI normal group and the mild group all returned to normal level, and one case in the moderate group and two cases in the severe group were remained abnormal. Sharma et al. (57) and Agrawal et al. (58) showed that BAEP improved in 77.2 and 76.5% children. This result indicated that, on the one hand, the BAEP abnormal rate of the patients with abnormal MRI was higher than that of the patients with normal MRI, and the BAEP abnormal rate of the patients with abnormal MRI increased with the increase of bilirubin level. It also shows that with the increase of bilirubin, the hearing damage is more obvious (59). On the other hand, it also proved the application value of MRI in neonatal jaundice patients, which could timely detect the abnormalities of central nervous system of the patients with jaundice, and provide imaging evidence for early diagnosis and early intervention.

Based on this study, we found that there were abnormalities in the neonatal brain's MRI and BAEP in the different bilirubin concentration levels. It indicates that in some conditions, even a low level of bilirubin concentration might lead to neurological damage in the neonatal period. So we strengthen the monitoring of neonatal jaundice and may use the MRI and BAEP to test the bilirubin induced neurological damage for neonataes.

## Limitations of the Present Study

Although the findings of this study indicate that in babies with jaundice, the brain MRI may show abnormal changes at mild and moderate levels of bilirubin, and there is no statistically significant difference between brain MRI abnormalities in the severe group, our results have been affected by a number of issues. First, we failed to measure the free bilirubin levels and internal environmental conditions such as serum albumin levels and pH values of the jaundice newborns participating in the study at that time, so it cannot be said with certainty that the baby's brain was exposed to bilirubin crossing the blood-brain barrier prime type. Secondly, the sample size in this study is relatively small, especially in the mild group and the group <2,500 g. Finally, the craniocerebral pathology test of newborn animal Jaundice model and long-term follow-up is lacking to validate our findings. This will be an issue that we need to address in this type of research in the future.

## Conclusion

In conclusion, in the presence of certain factors, such as premature, hypoproteinemia, and potential intrauterine hypoxia, central nervous system damage may also occur at low level of bilirubin and result in abnormality on MRI and BAEP. Meanwhile, MRI and BAEP can also be used to provide early abnormal information for the judgment of central nervous system damage to NHB neonatal patients without clinical manifestations of ABE, and offer clues for early treatment and early intervention so as to prevent the occurrence of severe brain tissue damage.

## Data Availability Statement

The original contributions presented in the study are included in the article/supplementary material, further inquiries can be directed to the corresponding author/s.

## Ethics Statement

The studies involving human participants were reviewed and approved by the Ethics Committee of the First People's Hospital of Taicang City. Written informed consent to participate in this study was provided by the participants' legal guardian/next of kin.

## Author Contributions

ZL: responsible for project writing/execution and paper writing. SD and FW: participated in project execution and data collection. HL: gave guidance on this subject and revised the paper. All authors contributed to the article and approved the submitted version.

## Conflict of Interest

The authors declare that the research was conducted in the absence of any commercial or financial relationships that could be construed as a potential conflict of interest.

## Publisher's Note

All claims expressed in this article are solely those of the authors and do not necessarily represent those of their affiliated organizations, or those of the publisher, the editors and the reviewers. Any product that may be evaluated in this article, or claim that may be made by its manufacturer, is not guaranteed or endorsed by the publisher.

## Abbreviations

ABE: acute bilirubin encephalopathy
TSB: total serum bilirubin
MRI: magnetic resonance imaging
NHB: neonatal hyperbilirubinemia
T1WI: T1-weighted imaging
T2WI: T2-weight imaging
BAEP: brainstem auditory evoked potential
ANSD: auditory neuropathy spectrum disorder
BIND: bilirubin induced neurological dysfunction
AN/AD: auditory neuropathy/dys-synchrony.
