# Corrigendum: Analysis on the MRI and BAEP results of neonatal brain with different levels of bilirubin

**DOI:** 10.3389/fped.2022.1042985

**Published:** 2022-10-25

**Authors:** Zhongxing Lu, Shouling Ding, Fen Wang, Haitao Lv

**Affiliations:** ^1^ Children's Hospital of Soochow University, Suzhou, China; ^2^ Neonatology Department, Changzhou Maternal and Child Health Care Hospital, Changzhou, China; ^3^ Pediatrics Department, Taicang First People’s Hospital, Suzhou, China

**Keywords:** MRI abnormality change, acute bilirubin encephalopathy, bilirubin, hyperbilirubinemia, BAEP

A corrigendum on Analysis on the MRI and BAEP results of neonatal brain with different levels of bilirubin by Lu Z, Ding S, Wang F and Lv H. (2022) Front. Pediatr. 9: 719370. doi: 10.3389/fped.2021.719370

In the published article, author Zhongxing Lu was missing an affiliation: “Children’s Hospital of Soochow University, Suzhou, China”. The author affiliation list has now been updated.

The authors apologize for this error and state that this does not change the scientific conclusions of the article in any way. The original article has been updated.

